# Affectivity, sexuality, and autism spectrum disorder: qualitative analysis of the experiences of autistic young adults and their families

**DOI:** 10.1186/s12888-023-05380-w

**Published:** 2023-11-17

**Authors:** Jordi Torralbas-Ortega, Judith Roca, Ruben Coelho-Martinho, Zaloa Orozko, Montserrat Sanromà-Ortiz, Victoria Valls-Ibáñez

**Affiliations:** 1 Hospital de la Santa Creu i Sant Pau and Nursing care Research Group. Research Institute Sant Pau (IIB SANT PAU), Barcelona, 08041 Spain; 2 Department of Nursing and Physiotherapy and Health Education, Nursing, Sustainability and Innovation Research Group (GREISI), Faculty of Nursing and Physiotherapy, University of Lleida, Lleida, 25199 Spain; 3 Health Care Research Group (GRECS), Biomedical Research Institute of Lleida, Lleida, 25198 Spain; 4 Department of Mental Health Nursing, Nostra Senyora de Meritxell Hospital, Escaldes-Engordany, AD700 Andorra; 5 Osona Salut Mental, Consorci Hospitalari de Vic, Vic, 08500 Spain; 6 Blanquerna School of Health Science, Ramon Llull University, Barcelona, 08025 Spain; 7 Departament of Nursing and Physiotherapy, University of Lleida, Campus Igualada, Lleida, 08700 Spain; 8 Health Center La Serra, Catalan Health Institute, Sabadell, 08208 Spain

**Keywords:** Autism, Autism Spectrum disorders, Sexuality, Affectivity, Families, Adolescence

## Abstract

**Background:**

Autistic people have communication, sensorial, and social difficulties, which on many occasions, make their adaptation on the sexual and affective levels difficult. For this reason, it is important to know the opinion of individuals with autism spectrum disorders (ASD) and their families, to offer this perspective to professionals to facilitate adapted health education programs in mental health units.

**Methods:**

This qualitative descriptive design presents the experiences of autistic individuals and their families in relation to the affective-sexual experiences from individual, family, and social perspectives. Two focus group sessions were held with eight family members and seven individual semi-structured interviews with autistic young adults. The transcripts were qualitatively analysed using content analysis.

**Results:**

Four themes (*Family and social dynamic*; *Social behaviour of the autistic individual; Affective-sexual relationships; Addressing affective and sex education*) and 13 related categories emerged from these results. Communication and social interaction problems act as barriers for young adults when developing affective-sexual relationships, leading to the emergence of negative feelings and experiences that reinforce avoidance behaviours, further intensifying their difficulties in interacting with others. Families, especially mothers, exhibit a poor perception of their ability to provide affective-sexual guidance, leading to anxiety and frustration. There are also reports of poor sex education and lack of support systems.

**Conclusions and implications for practice:**

The experiences of young people and their families are sometimes conflicting when it comes to affectivity and sexuality, but the parental role emerges as relevant in the sex education process. Families play a pivotal role in sex education, which is why professionals should provide them with support and information through health education programmes, foster empathetic communication and promote sexual and emotional development that is adapted to the characteristics and interests of autistic people.

**Supplementary Information:**

The online version contains supplementary material available at 10.1186/s12888-023-05380-w.

## Introduction

Autism spectrum disorder (ASD) is a neurodevelopmental condition that is typically characterised by difficulties with social communication along with repetitive behaviours and specific interests, difficulties in adapting to unexpected changes, and an atypical sensory sensitivity profile [1]. ASD occurs in 1.23% of the child and adolescent population in Spain and is diagnosed in a higher proportion of males than females [2]. This gender bias in the diagnosis has been tried to be explained by various theories, centered on the deviation of the diagnostic tools that exist towards the male presentation of ASD, the greater social skills of women and the greater capacity for camouflage of women (play more calmly, more timid, etc.) [3].

Socialisation is one of the key factors in promoting healthy learning of behaviours in all areas of life. Therefore, a key point of care at family level for autistic individuals is socio-affective learning [4]. Socialization is one of the key factors in promoting healthy learning of behaviors in all areas of life. Therefore, a key point of care at family level for autistic individuals is socio-affective learning [4]. Affectivity is an emotional dimension that involves the feelings, emotions and attitudes that a person experiences and expresses towards themselves, towards others and towards the world around them. It is a fundamental and complex aspect of the emotional life of human beings and plays an important role in our interpersonal relationships, decisions and behaviors. Affectivity is closely linked to sexuality, as an expression of social behavior, within a continuum that includes friendship, romantic friendship and sexual expression [5].

Autistic young adults are often infantilised and viewed as asexual or as lacking interest in intimate relationships [6, 7]. However, recent research [8–10] indicates that autistic individuals desire and seek out affective and sexual relationships and engage in a variety of sexual behaviours typical of most young people. As such, addressing affective-sexual relationships is crucial for improving the quality of life of autistic people and their families, as they are at high risk of negative experiences, such as sexual abuse, loneliness, unwanted pregnancies, sexually transmitted diseases, and inappropriate sexual behaviour [10–12].

Several authors [13] highlight that adolescents and autistic adults have limited knowledge and awareness of sexuality. Sex education facilitates their adaptation, but the characteristics of the disorder itself, such as rigidity in thinking and lack of flexibility with established norms, may hinder their adaptation process [11, 14]. Families play a vital role in fostering healthy sexuality and affectivity, in supporting young people’s decision-making, in helping them to overcome the challenges of adolescence and move into adulthood [4], and in monitoring and minimising the risks to which autistic individuals are exposed due to their vulnerability. Important difficulties have been described in the adaptation of autistic people at the level of affective and sexual behavior with important consequences, focused on identity and sexual tendency, social and romantic behavior, and inappropriate or inadequate sexual behavior, as well as their main concerns [15]. Education and health professionals can be allies in supporting this family intervention, as they recognise the key role of families in sex education [16]. In order for education or a training intervention to be successful, the various parties involved must be closely interconnected [17]. One of the main studies that have been published is the Tackling Teenage Training (TTT) [18], it is based on joint work (person, family and professionals), the development of communication skills and the approach regarding topics related to puberty, sexuality and intimate relationships. These promising programs have not yet been extended and culturally validated.

Therefore, the main objective of this study was to explore the experiences of autistic people and their families in relation to affectivity and sexuality from individual, family, and social perspectives.

## Methods

### Design

This study used a qualitative descriptive design [19, 20] that seeks to provide insight into the study phenomenon without preventing interpretation of the data. Interconnected interpretative qualitative practice [21], in this case involving the experiences and perceptions of young adults diagnosed with ASD and their families, can contribute to improving aspects of education and professional practice.

This research is part of a larger study which seeks to improve affective and sexual education in mental health units, one of the phases being to explore the perception of autistic people and their families. It is based on the foundation that patients are willing to participate in health education when their perceptions and beliefs are adequately addressed [19]; therefore it is essential to explore them.

### Setting and participants

The study was carried out at the Consorci Corporació Sanitària Parc Taulí in Sabadell, Barcelona, Spain, which is a tertiary healthcare facility that is part of the Catalan Health Institute care network, currently attending to a population of 493,000 citizens. This Mental Health Area cares for individuals with a range of psychological conditions, whether in the child, adolescent, or adult populations.

Family members were invited to participate in the focus group sessions and the autistic young adults took part in semi-structured interviews. In both cases, purposeful sampling was used. Information-rich participants that could cover a wide variety were identified [22]. The criteria were the ability to participate, the level of support, and their orientation and sexual tendency in order to obtain diversity. The recruitment of participants was done by telephone. A total of 8 participants were included in two separate focus group sessions, and 7 individual interviews were conducted until data saturation was reached. A stopping criteria was established when no new categories appeared in the last two interviews or the last focus group that was analysed [23]. There was no family relationship between autistic individuals or between autistic individuals and family members.

The focus groups included members of families in which the adolescent or young adult had been diagnosed more than 5 years prior to the study. The research team considered that in a period of at least 5 years, the families would have a sufficiently relevant experience to provide information of great interest and variety to the research. These family members were biological parents, adoptive parents, or guardian relatives, and no exclusion criteria were established. The family members included 7 women and 1 man between 47 and 59 years of age. Only two family members were permanently incapacitated for work; the others were in stable employment. All families had more than one child in their care. A total of 6 relatives refused to participate, giving reasons such as lack of time or difficulties in talking about the subject.

The inclusion criteria for autistic individuals in the semi-structured interviews were: being diagnosed with ASD more than 10 years, being an adolescent or a young adult (aged between 14 and 27 years old) and have a degree of linguistic competence that would allow for communication. This minimum time of 10 years after diagnosis was considered sufficient by the research team, to obtain in-depth information and rich in experiences, that could ensure the criteria of scientific rigor of the proposed study.

Autistic people who were assessed by their therapist as a level 3 support (defined in the Diagnostic and Statistical Manual of Mental Disorders, 5th Edition; ASD Level 3: requiring very substantial support, characterized by severe challenges in social communication as well as extremely inflexible behavior and other difficulties) were excluded, as it would have prevented smooth communication. For evaluation and diagnosis, the autism care team specializes in administering the ADOS-2 (Autism Diagnostic Observation Schedule) and/or the ADI-R (Autism Diagnostic Interview-Revised) after the initial assessment and clinical interview with your referred therapist. Nine interviews were conducted, but two were discarded, as the interviewees responded only in monosyllables or kept quiet. None of the participants refused to participate in the study. Consistent with the higher prevalence of ASD among males, interviews were collected from a total of 4 men, 2 women, and 1 transgender man, aged between 20 and 27 years old. Four of them had never had a partner or sexual relations, two had a partner—but only one had had sexual relations—and one did not have a partner but had had sexual relations with female sex workers. The characteristics of these participants are presented in Table 1.

**Table 1:** Characteristics of the sample (autistic individuals)

ID	Relational Status	Age	Gender	Level of support	IQ	Living situation	Education/ Word	Comorbidity	Sensitivity
01	SINGLE	22	M	2	AVERAGE 91	With parents	STUDENT	ADHD	H
02	RELATIONSHIP	20	F	2	LOW 76	With parents	STUDENT	-	h
03	SINGLE	22	M	1	AVERAGE 104	With parents	STUDENT	-	N
05	SINGLE	27	M	1	SUPERIOR 110	With parents	STUDENT	ADHD	H
07	SINGLE	20	FTM	1	AVERAGE 101	Group home/ With parents	UNEMPLOYED	ADHD	H
08	RELATIONSHIP	21	F	1	SUPERIOR 121	With parents	EMPLOYED/ STUDENT	OCD	H
09	SINGLE	23	M	2	LOW 83	With parents	STUDENT	-	N

**ID**: Identification; F: Female; M: male; FTM: Female to Male; **Level of support**: 1 (requiring support), 2 (requiring substantial support), 3 (requiring very substantial support); **IQ**: intelligence quotient and level of intellectual development according to Wechsler scales assessment (WISC or WAIS); **Comorbidity**: ADHD (Attention Deficit Hyperactivity Disorder), OCD (Obsessive Compulsive Disorder); **Sensitivity**: H. Hypersensitivity, h: hyposensitivity, N: Neutral

### Data collection

Focus groups and semi-structured interviews were used as the main data collection techniques. The thematic script that guided the groups and interviews contained questions introducing the study dimensions that were defined based on both the reviewed literature and the experience of the research team. On the one hand, a narrative search was carried out to explore the theme with keywords such as “Autism”, “Sexual*” and “Affectivity”. On the other hand, the professional and scientific experience in mental health issues, autism and qualitative methodology of the research team allowed the detection of key dimensions. Therefore, the dimensions to be explored were: (1) adolescence and family, (2) behaviour and interpersonal relationships, (3) affective-sexual education and training, and (4) affectivity and sexuality as experienced by autistic people. These 4 dimensions were introduced in the questions posed to the two groups. Table 2 shows the schematic script for the focus group sessions and individual interviews.

**Table 2:** Dimensions and associated questions

Dimensions	Questions for FAMILIES	Questions for AUTISTIC PEOPLE
Adolescence and family	What was or is adolescence like for your child? And for you as a family?	What was your adolescence like? How did your family and the people around you help you?
Behaviour and interpersonal relationships	How does your child relate to others? What aspects have you worked on to foster the most adaptive behaviours? Has your child had any romantic relationships or sexual experiences?	How do you deal with relationships with other people? How do you make and maintain friends? Have you ever had a romantic relationship with anyone?
Affective-sexual education and training	How have you worked on the subject of affectivity and sexuality with your child? What problems have you identified? How have they been addressed?	How did you learn to relate better with people? Do you think you know a lot about sexuality? Where did you learn all this?
Affectivity and sexuality as experienced by people with ASD	Do you think that young people diagnosed with ASD have different affective and sexual behaviour than other adolescents? What concerns do you have in this respect? What support do these children have or should they have to be able to develop in this area?	Has anything unpleasant or unwanted happened to you while in a romantic or sexual relationship? What concerns you in these relationships? Who helps you when you have questions or problems?

After contacting the participants in person, appointments (time and place) were made for each interview and focus group. It should be noted that, at the request of the participants, three interviews were conducted in a hospital room away from the mental health facility, another three were conducted in the participants’ own homes, and one interview was held in a room at the secondary school where the participant studied. Interviews lasted between 35 and 60 min. The focus group sessions were held in rooms adjacent to the hospital, away from the mental health area. The session with the first group lasted 70 min and the session with the second group lasted 90 min.

The focus groups were led by two qualitative research experts who were not involved in the study, and the interviews were conducted by two members of the research (J.R., V.V-I.) team who were not acquainted with the people with ASD. At the time of the research, the PI was a nursing assistant in the mental health area of the XXX. The two data collection strategies took the same approach: after thanking the participants for their willingness to participate, they were informed that the conversation would be audio-recorded (no images) and that they had to sign the informed consent form to be included in the study, which they did. Additionally, field notes were taken in order to complement the transcriptions.

The interviews and focus group sessions were conducted between May and September 2019. All observations and incidents that occurred during the interviews and focus group sessions were documented in a field notebook and incorporated into the transcripts.

### Data analysis

The research team performed the content analysis of the data following the guidelines proposed by Graneheim et al. (2004, 2017), using the NVivo v12 software. The information from the interviews and the two focus groups was transcribed verbatim. Reading and re-reading the transcripts allowed us to identify the units of analysis. Then, a coding structure was created, in which codes, categories and themes were established on the basis of the reoccurring ideas. This structure was systematically applied to the 9 transcripts (2 focus groups and 7 interviews). Data analysis was performed inductively based on the dimensions of the data collection script (Table 1).

## Criteria of rigor and quality

The researchers worked systematically on quality and rigour (credibility, reliability, and transferability) [24, 25] using triangulation, (transcripts from relatives and people with ASD), data saturation (degree of new contributions in relation to the previous ones) and validation by informants of the information. Participants accepted the transcripts and provided no further information. The transcripts were then verified and analysed by two independent researchers to improve credibility and, finally, the thematic areas defined in each analysis were discussed and agreed upon by the whole research team in a joint working session. This study also adheres to the Consolidated Criteria for Reporting Qualitative Studies (COREQ) checklist [26]. The research team validated the saturation of the data with the number of participants used, by identifying the redundancy of the information in the data analysis (categories and themes) [27]. Additionally, the final report contrasted the coherence and clarity between the data collected and the findings.

### Ethical considerations

This study was assessed and approved by the Clinical Research Ethics Committee at the Parc Taulí Hospital, with reference number 2018-509. The participants signed the informed consent in which they were explicitly informed about the objectives of the research, about their voluntary participation and the possibility of not releasing their data or withdrawing from the study. The anonymity of the informants was respected at all times. Each participant was assigned an alphanumeric code. All names that may have identified persons associated with the autistic individuals and/or their families were removed from the text. The recorded documents and transcripts are secured and only accessible to the project’s principal investigator.

## Results

**Fig. 1:**
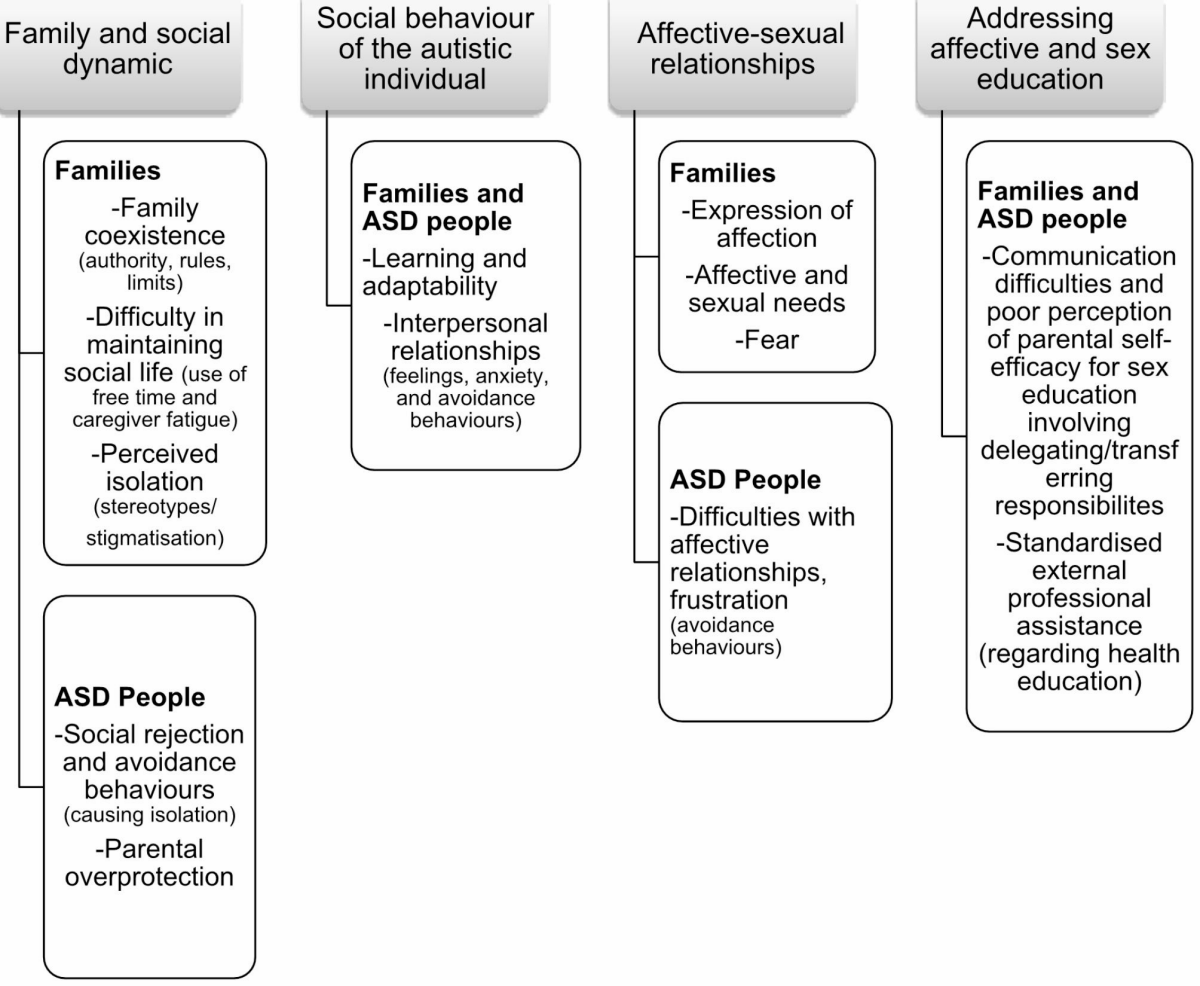
Themes and categories identified in persons with ASD and their families

Two initial, more generic themes are drawn from the results: *Family and social dynamic* and *Social behaviour of the autistic individual*. These cover aspects of familial, social, and behavioural coexistence that are directly related to affective-sexual aspects. They are more general in nature, but allow the categories to be viewed from the perspective of the participants in a continuum between social and affective behavior. Then, the *Affective-sexual relationships* theme is identified, which refers to the need for interaction and socialisation; and finally, the theme *Addressing affective and sex education*, which analyses aspects of dealing with affectivity and sexuality and shows the difficulties of young people and their families in obtaining information and support. From these four themes, 13 related categories emerge, distributed by themes and participants, as outlined in Fig. 1. It should be noted that units of meaning are presented in the text for the two types of participants explored: families (identified by an F, followed by the participant number and the number of the focus group, G1 or G2) and autistic people (identified by a J and the participant number).

### Family and social dynamic

Families agree that living with an autistic individual has an impact on the dynamic of the family nucleus, with very complex situations arising from everyday and apparently simple activities that can disrupt aspects such as intimacy and family structure, causing family exhaustion.

*‘He just lives his life. I mean, he doesn’t care whether or not there’s someone in the bathroom…’ F2G1*.

*‘I don’t have a private place, so to speak’ F1G1*.

*‘Our son runs, falls down, pushes us, cries, breaks things, and then of course, we have two younger daughters…’ F2G1*.

*‘…To think that it’s been 24 years without a single moment of respite, without a good night’s sleep, without having anyone to leave him with.’ F2G1*.

However, they also maintain the need to educate their child in behaviours that should be kept in the private sphere to ensure harmony in the family nucleus.

*‘The same goes for touching his penis. Well, he can’t do it in the dining room. Not in the dining room. He has to do it in his room or in the bathroom. We’ve made that clear. Yes. He goes to his room… he doesn’t close the door, of course, but he does it in his room.’ F1G2*.

*Parents’ social lives* can be severely impacted, separating them from their social and family environments. Even when they do find time for themselves, it is not without stress.

*‘I need to find myself a hobby because there are times when I’m climbing the walls. And I leave him alone. I go to painting lessons… I don’t know what he might be doing while I’m there. Sometimes the neighbours tell me that he goes out on the veranda and starts saying things…’ F3G1*.

*‘[My partner and I] go shopping on Saturdays. And the [mobile] phone doesn’t stop [ringing]. He keeps bothering us…’ F2G1*.

*‘It holds us back a bit… If he doesn’t want to go where we want to go, then end of story…’ F1G2*.

Part of the family’s perceived *isolation* is caused by the rigid way of thinking of the autistic individual, by the feelings generated by their behaviour in public and, above all, by their lack of understanding of how social interactions work.

*‘We had to go out to the beach and tell him, ‘Go pee in the water, it’s OK’, but we had to go back to the flat so he could pee. He won’t pee in a bar.’ F2G1*.

*‘We were working on how to take a train to go to a wedding, we were working with the therapist, with the doctor, with the psychiatrist, with everyone. But when five of us arrived at the station, my husband had to stay with him, he wouldn’t get on the train.’ F2G1*.

*‘…He was different. I was ashamed of his behaviour.’ F4G1*.

*‘[For example,] we get on the bus and he starts crying… and someone says, ‘your son is too old [to be crying like that]’… and things like that.’ F3G1*.

*‘…Everyone rejected us. I mean, my son would never be invited. The other parents wouldn’t talk to me.’ F2G1*.

In contrast, autistic individuals identify this *social rejection* and how it varies depending on the moment and is persists over time in other shapes or forms (isolation, aggression, indifference, etc.). They are also affected by judgmental comments made by ‘outsiders’ who are unaware of their situation.

*‘I had almost no friends and they always picked on me a lot, too. So, I always shy away from people a bit.’ J2*.

*‘…Well, when I started university, people… the situation changes a bit and people stop attacking you physically… Even so, you get what I call ghosting… they give you the cold shoulder.’ J5*.

*‘Because there’s a lot of people asking me ‘What is ASD?’ And I have to explain it to them… [but] they weren’t interested at all.’ J5*.

These autistic youngs do not perceive any conflict within the family nucleus, and only a few comment on their parents’ *overprotectiveness* as a limiting element, particularly in the case of their mothers.

*‘My mother tends to protect me and that means that sometimes I don’t want to tell her very much…’ J3*.

*‘…When bad things happen to me, I don’t tell my mother because I don’t want to make her suffer. I mean, I don’t know why I do it, but it just comes out that way.’ J8*.

This limiting view of overprotection is shared by their mothers, but despite being aware of it, they maintain it.

*‘I [must admit that] I also spoil her because I do everything for her.’ F3G2*.

### Social behaviour of the autistic individual

Families recognise that their children have a high capacity for *learning* but restricted interests and limited *adaptability*. On the one hand, despite their intellectual diversity, these individuals show great interest in certain subjects. On the other hand, they are also limited by their lack of interest in other topics and problems inherent to the disorder, such as sensory hypersensitivity and difficulties in understanding social interactions. Something that families and autistic people both find very stimulating, and value very positively is when they are treated well.

*‘Another positive aspect is that you don’t have to teach him about computers and electronic gadgets.’ F3G1*.

*‘…things I don’t like bore me a lot.’ J9*.

*‘Well, it’s just that, I attended [secondary school] but I had a very bad time there with teachers and the other students, and when [my classmates] started shouting a bit or something like that, I used to get very anxious and couldn’t control myself, it was horrible. The same with teachers when they misunderstand things, ugh, you get so nervous that it’s just ugh…’ J2*.

*‘…I’ve felt more comfortable because they’ve been nice to me, you know? They’ve been really nice to me and they’ve treated me the way I feel I want to be treated.’ J7*.

Regarding the establishment of *interpersonal relationships*, families identify several behaviours that interfere with forming social relationships, such as unfiltered honesty, face value acceptance, lies, obsessiveness, etc.

*‘…They have a problem relating to others. But when they do interact with others, they say it exactly as they see it, whether you like it or not’ F4G1*.

*‘He always accepts one person. If he doesn’t like that person, he’d isolate himself and won’t have anything to do with them.’ F2G1*.

*‘What I mean is that my son lies, but he does it in such a way that the person on the receiving end will believe him.’ F2G2*.

*‘All his friends, boys and girls, are younger than him. He never seeks the friendship of those older than him.’ F4G1*.

*‘He gets obsessed very quickly with things. Whether it’s some random thing, whether it’s a boy he likes, whatever.’ F2G2*.

Families acknowledge that part of the conflict that they experience is caused by others (outsiders) but also by their children’s own differential characteristics.

*‘… He got on well with all the children at school, when he was in primary school. Of course, because of his condition, he had problems with some children, but it was because they were picking on him.’ F4G1*.

*‘It’s just that, as he says, he’s overwhelmed by children, you know? He says: ‘They annoy me.’ That’s why he doesn’t want to [interact with them].’ F3G1*.

A number of young adults diagnosed with ASD have learned to maintain a minimum number of acceptable social relationships and to even recognise what may be holding them back. In contrast, social interaction with others is a source of anxiety for other autistic young individuals, for whom self-isolation does not constitute a problem.

*‘It’s just that I’ve been learning. Before, I couldn’t, I just couldn’t do it. And my mind would go blank, and I wouldn’t know what to say.’ J2*.

*‘But actually, they say that being alone is overwhelming, that it puts you in a bad mood, that nobody can be alone… but I’ve stayed at home for several years so happily without going out. I have saved myself a lot [of trouble] by not going out.’ J9*.

*‘My friends think I’m antisocial. I can definitely socialise with people, but I prefer to be in my bed, at home.’ J1*.

All young people reported having a very small circle of friends, with some only identifying one person as a friend. One of the main characteristics they emphasise in their friends is that they treat them well.

*‘[the fact that he/she] looks after me…’ J9*.

*‘…who makes me feel more confident, who gives you that confidence so that you can express what you might find more difficult to express.’ J3*.

### Affective-sexual relationships

One of the characteristics of autistic individuals is their difficulty in expressing their *feelings* in a neurotypical manner. However, they express them in other ways that the people closest to them can interpret. Even autistic people recognise this difficulty.

*‘If he sees a person he likes, he’ll start laughing, but he won’t say ‘I like [him or her]’ or express his feelings.’ F2G1*.

*‘When I’m sad, I can’t cry… It comes but then it goes away.’ J9*.

This difficulty becomes more apparent when they fall in love, as they feel the attraction but cannot explain it. It is an experience and a necessity at the same time.

*‘He’d tell me ‘Mum, I like Mireia’, and I’d tell him ‘You like her a lot, don’t you?’ and he’d say ‘Well, I obviously feel something.’ F4G1*.

*‘Does he understand [the notion of] falling in love? I don’t know… He’s been telling me lately that he wants a girlfriend to fall in love with.’ F3G1*.

Parents’ thoughts about their children’s *affective and sexual needs* is ambivalent: they recognise the changes of adolescence in their children but still see them as children, with some parents who don´t initially identify that their children have such needs.

*‘On the one hand you see that he’s a teenager and you see that he has those needs, but on the other hand you see that he’s just a child. He comes to you with stuffed animals, to play on the sofa, you know what I mean? He’s very childlike. But of course, on the other hand, you see that he has that teenager attitude, of course.’ F1G2*.

*‘My child doesn’t have such needs.’ F4G1*.

*‘He now has the habit of touching himself, but it’s not because he wants to masturbate or anything, it’s just a tic.’ F2G1*.

Affective and sexual feelings and needs arouse parents’ *fears* for their children, for their own safety and for the safety of others. They also trigger overprotective behaviours and considerable uncertainty about the future.

*‘Well, I do worry about someone hurting him… forcing him to do something he doesn’t want to do. He’d answer ‘Yes, yes, yes’. He seems to understand those things, he seems to… but then again you don’t know to what extent he does.’ F1G2*.

*‘Then we talked to him, we told him: ‘You can’t do that.’ And also, well, you know, that all those things require the girl’s consent and [be done] in places that aren’t, that aren’t, you know, places with people or where you can be seen…’ F1G2*.

*‘[I’d always tell him:] ‘Be careful, you will be disappointed’. I’ve always tried not to get his hopes up.’ F4G1*.

*‘I just can’t imagine him having a partner. I don’t know if I can’t imagine that now because I see him as very childlike and stuff… Maybe he may have one in the future, a partner who is also at home… I just don’t think about it. I haven’t thought about the future.’ F1G2*.

Young adults reported difficulties in interacting with others when interested in someone else, sometimes alluding to lack of control or to a poor understanding of the situations influencing socialisation and successful relationships. They also reported their willingness to form relationships with other people with and without disorders.

*‘I’ve had very frustrating relationships. I’ve never had a girlfriend or… I’ve fought hard for a lot of women, but they’ve always said no. And I’ve suffered a lot… I’ve tried to be with them and they’ve always felt harassed or had had a hard time with me.’ J5*.

*‘I have fallen in love, but I have regretted it because in the end you feel it for a person you love very much… but that person is more focused on their education…’ J3*.

*‘Any relationship can fall apart in a matter of seconds, so sometimes it’s difficult, I can’t control that.’ J2*.

*‘I know many people who have partners, but they are usually partners who have either Asperger’s or other disorders…’ J5*.

Young people diagnosed with ASD feel more challenged when addressing gender identity issues.

*‘I am a trans man, it’s not because of any interest [I may have], it’s because I really feel it, because I experience it that way from a very early stage of life, very young, very early… I’ve never felt the way I am biologically.’ J7*.

### Addressing affective and sex education

*Communication* between parents and children is often two-way and can be complex for a variety of reasons (lack of acceptance, not recognising the child’s needs, lack of understanding, etc.). Mothers are often the ones who try to address communication issues, sometimes receiving a refusal to do so or having personal difficulties in addressing them.

*‘I didn’t have to explain anything to my son, because whenever I tried to explain these things to him, he’d say ‘Mum, you don’t have to explain anything to me, I already know,’ or ‘Come on, mum, I know more about sex than you do’, so I don’t have to explain anything to him.’ F4G1*.

*‘No, either he doesn’t want you to explain anything to him or… we don’t know how to do it properly.’ F1G2*.

*‘I can’t seem to be able to… I don’t know why, I can’t seem to be able to explain anything to him….’ F3G2*.

In this process, family members seek *external professional help* to address these issues, and some even only talk to professionals when an uncomfortable or conflictive situation of an affective or sexual nature has occurred. Moreover, families believe that healthcare professionals are the most suitable professionals to deal with the subject.

*‘We told his psychiatrist, his doctor, about this situation and I said: ‘Look, this is what has happened, doctor.’ F2G1*.

*‘Well, I told the psychiatrist, and the truth is that she explains it in a way that… is much better than how we do. The last time I liked it a lot because [name] was very attentive when she was explaining things to him… It’s better that people outside the family explain things to him rather than us.’ F1G2*.

This view of healthcare professionals as supportive and helpful is shared by the young respondents. They admit to having talked to professionals when they had problems, but they do not always know how to take that step.

*‘I get coital headaches. The neurologist gave me some pills to help me stop them, but sometimes they worked and sometimes they didn’t.’ J8*.

*‘I don’t feel anything when I masturbate… But how do I tell him [the doctor]? He’s very nice, but I wouldn’t dare tell him. He hasn’t asked me.’ J1*.

Young people also perceive formal sex and affective education as insufficient for their development, although parents tend to trust that this source of information is sufficient.

*‘Yes, like for example in secondary school, when they came to teach you about sex education, the only thing they showed you was how to use a condom. But I mean, there’s more to it. It’s not just that and STDs.’ J2*.

*‘They have explained it at school.’ F4G1*.

*‘This subject… He worked on this when he was in special school. And at the occupational facility, too.’ F2G1*.

## Discussion

This study explores the experiences of autistic people and their families in relation to the sexual affective experience from an individual, family and social perspective. The findings of our research illustrate an experience that is, at times, shared by autistic individuals and their families, and, at other times conflicting. Sex-affective relationships are unique and have their own characteristics, but are influenced by other forms of relationship and personal, family or social factors. Therefore, general aspects of family dynamics and social behaviour, and specific aspects of sexual- affective relationships emerged, so the discussion is presented in two sections.

### Experience of family and social relationships

Parents define adolescence as a time of great complexity for coexisting with their children, as it is difficult to maintain daily routines and personal privacy. Any activity, however simple and commonplace it may be, can become a source of conflict. However, the young people interviewed did not mention conflict at home, instead recognising the family environment as a safe and trusting space. Family members experience high levels of anxiety, although they keep actively working on their children’s autonomy and behavioural appropriateness to facilitate their children’s transition to adulthood in an autonomous way [28]. As in other studies [29], our results show an increase in maternal control and overprotectiveness at this stage, which, based on the findings of this study, results in young people engaging in avoidance behaviours or restricting the information they provide to their mothers.

Young people diagnosed with ASD and their family identify situations of social rejection in different settings such as school and how they are maintained in adulthood. Misunderstanding and ignorance of ASD often translates into avoidance behaviours. Other authors [30] confirm these findings in multiple studies. Negative experiences such as these are a reason for the isolation of young people with ASD, although they recognise that they feel more comfortable in their solitude. Young people consider friends to be those who help them and treat them well, showing persistent interest in affective relationships and friendships, as other authors agree [7], with a high degree of satisfaction in them [31]. Autistic young people and their families alike recognize the emotional wellbeing generated by appropriate social interaction.

In addition, the study findings not only show the learning capabilities of autistic young people, but also their narrow topics of interest. These topics of interest may be a good avenue for communication, but social relationships may be quite constrained if they do not find other people who share the same interests, resulting in a loss of interest in any other topic of conversation [32, 33]. Moving on to feelings and affective-sexual relationships, these difficulties with communication and social interaction represent both intra- and interpersonal barriers [7] while decreasing their opportunities to obtain sex-related information from their classmates and peers [34]. Similarly, participants commented on limitations in learning social norms and recognised the lack of filters as sources of difficulties with social interaction, with establishing affective relationships and of a decreasing contact with peers. These limitations can be mitigated in good school and family environments, where managing these conflicts can be considered as part of the learning process, as is stated in the systematic review of André et al. [4].

### Experience of affective and sexual interaction

Family members sometimes experience ambivalent feelings about their children’s affectivity and sexuality, which, as reported in other studies [34], may lead them to dismiss their children’s sexual experiences. In addition, they indicate that it is very difficult for them to teach their children about affectivity and sexuality, given that most young people are reluctant to discuss this topic with them or because of the limitations they expressed, such as lack of knowledge on the topic [4]. However, the literature shows that it is advisable to address this issue in order to avoid many of the risks faced by autistic young people [4]. Therefore, this awkwardness or lack of two-way communication causes parents to experience feelings of stress, thus impacting their perceived self-efficacy as educators. As such, a number of studies show a negative relationship between stress and perceptions of their own abilities, particularly among mothers of children diagnosed with ASD [35]. Consequently, they leave training in this area in the hands of health or education professionals. Although sexual education in our society in general happens indirectly, is silenced or impregnated with a metaphorical language that makes it difficult for autistic people to access knowledge [36].

In addition, sex and affective education in schools is perceived by young people as inadequate, as it ignores relational aspects, which are the ones in which they have more difficulties and which make them more vulnerable [36]. Therefore, parents, educators, and healthcare professionals should provide the opportunity to discuss this topic with an entirely open-minded attitude [37] and within the limits of a biopsychosocial framework [10]. A recent qualitative study [16] described the difficulties experienced by professionals during their training to be able to address affectivity and sexuality in autistic individuals. Even though those professionals recognised the importance of such issues and their role in addressing them, the problem is compounded by the fact that the young adults participating in this study recognised that they did not discuss sexuality issues with professionals, in line with other studies [37]. The establishment of programs specifically dedicated to autistic people, which address sexuality and affectivity in a direct and multifocal way, in which professionals, family members and autistic people participate, would facilitate adaption of the sexual-affective level, which normally causes a lot of anguish and confusion. Multiple proposals have been raised that must be validated in the different groups of autistic people, adapted to their main characteristics and the poor sexual and affective education they receive dus to their complex social relationships taken into account [15, 18].

Interest in sexuality varies among autistic young people. The visión of sexuality is not homogeneous even in the small group of participants explored in this study. As in the qualitative study by Cheak-Zamora et al. [37] there are participants with (4 in our study) and without an interest in having relationships. Those who are interested detail uncomfortable, frustrating relationships with problems to interact. Another element that emerges is the type of partner and the willingness to relate to people without autism. In general, they view sexuality as a necessity. However, when problems arise, they feel little support and have too many difficulties in discussing them. It should be noted that affective or sexual relationships are often both difficult and desired [7], especially among females who have more experience in this area, less interest in intimate interactions, and a higher risk of victimisation [38, 39]. A number of authors [40] also report these feelings in gender-non-conforming autistic women and recommend that gender identity and sexual diversity should also be emphasised. As such, establishing a reliable support network would make it easier for them to express their fears, concerns, and difficulties on the path to full affective expression [41, 42].

Perhaps the most important challenge for professionals supporting autistic individuals and their families is to establish specific, culturally-adapted programmes to assist with the condition and ensure universal access to sexual health education [43]. For family members, as in the study by Dewinter et al. [44] the problems between couples are easier to address as they are related to safe sex, preventing abuse, privacy and posible prevention of pregnancy as opposed to other aspects of sexual health such as sexual arousal or those related to pleasure such as masturbation. And for people with autism, it should be noted that the reduced knowledge and a lack of effective communication (families and professionals) combined with social difficulties that emerge in this study, can lead to inappropriate behavior and denail of sexual health as part of a healthy and satisfactory life [45]. The aim should be to provide comprehensive sex education which includes parental communication about sexuality, expectations, and sexual experiences, both alone and with a partner, in order to have a healthy sex life and prevent negative experiences [46].

### Limitations

Some limitations of the study are inherent to the qualitative designs and the type of purposeful sampling. In addition, it should be noted that since the simple is from a single center, all the participants (relatives and autistic people) belong to the same mental health unit and therefore receive the same care. The possibility of unifying some lines of action in affective sexual education between different care centers would allow for a more extensive analysis, as well as the combination with possible quantitative data. Finally, this study has a descriptive approach; therefore, subsequent studies that delve into the analysis of comprehensive sexual health would be of great help for interventions to be carried out by health professionals.

## Conclusions and implications for practice

The experiences of young people and their families are sometimes conflicting when it comes to affectivity and sexuality, but the parental role emerges as relevant in the sex education process. Firstly, both groups recognise the emotional wellbeing that arises from appropriate social interaction. Secondly, communication and social interaction problems act as barriers for autistic young people when developing affective-sexual relationships, although interest in sexuality is variable in this group. Thirdly, this group has a high learning capacity which is restricted only to their topics of interest. Young adult participants report poor education, problems with and lack of communication with professionals, as well as inadequate support systems, while parents do not feel capable of providing the sex education that their children need.

Finally, families have a central role to play in providing sex education to their children, and therefore, professionals should provide them with support and information to foster communication and trust. That is why specific, adapted health education programs which comprehensively address this issue are needed in the area of mental health. In order to achieve smooth sexual and affective development among young people with ASD and increase their perceived individual autonomy in relation to their sexual and affective needs, specifically adapted health promotion programmes are required. At the familial level, acceptance of the uniqueness of neurodivergence should be improved among families, who should also be empowered to play their crucial role in their children’s affective and sexual health education. On a social level, environments rich in communal experiences should be promoted as cohesive elements that contribute to the acceptance of ASD, while avoiding the stigmatisation of individuals with the condition.

### Electronic supplementary material

Below is the link to the electronic supplementary material.

Supplementary Material 1: COREQ Checklist

## Data Availability

Please contact the corresponding author (judith.roca@udl.cat) to request data.
